# Unseen Threats of Chronic Diseases among the Middle-Aged: Examining the Feasibility of Well-Defined Healthcare Vouchers in Encouraging Uptake of General Checkups

**DOI:** 10.3390/ijerph191811751

**Published:** 2022-09-17

**Authors:** Jasen Kin-Fung Leung, Martin Chi-Sang Wong, Eliza Lai-Yi Wong

**Affiliations:** 1 JC School of Public Health and Primary Care, Faculty of Medicine, The Chinese University of Hong Kong, Hong Kong SAR, China; 2 Centre for Health Education and Health Promotion, JC School of Public Health and Primary Care, Faculty of Medicine, The Chinese University of Hong Kong, Hong Kong SAR, China; 3 Centre for Health Systems and Policy Research, JC School of Public Health and Primary Care, Faculty of Medicine, The Chinese University of Hong Kong, Hong Kong SAR, China

**Keywords:** healthcare system, health promotions, health voucher scheme, disease prevention

## Abstract

***Background:*** The ageing population and the emergence of chronic diseases continue to pose immense challenges to the healthcare system. This study aims to explore how likely middle-aged citizens could be encouraged to attend health checkups by well-defined healthcare vouchers, and to explore potential factors associated with the uptake of health checkups. ***Methods:*** A cross-sectional survey with self-administered structured questionnaires was conducted among Hong Kong residents aged 45–59. The questionnaire consisted of 25 items, including attitudes toward healthcare vouchers and checkups, utilisation patterns of healthcare services, and socio-demographics. ***Results:*** We received 278 responses between June and September 2021. Among the study participants, 62.6% (174) attended regular checkups currently, and a total of 252 (90.6%) indicated that it was likely for them to undertake health checkups with well-defined vouchers. This proportion showed an increase of 44.8% after introducing vouchers (78 of 174) when compared with the proportion currently attending regular health checkups. Multiple logistic regression analysis revealed that the perceived barrier of health checkup uptake included financial cost (AOR 0.367, 95% CI 0.162–0.832, *p* = 0.016), whilst the government’s recommendation (AOR 1.685, 95% CI 1.052–2.698, *p* = 0.030) and full support by the employer-purchased insurance (AOR 2.395, 95% CI 1.036–5.523, *p* = 0.041) were positively associated with uptakes. ***Conclusions:*** Financial cost is a significant barrier to health promotion and disease prevention. Well-defined vouchers, as a demand-side financial tool, were widely accepted by our participants as incentives to undergo health checkups. Our findings indicate that the voucher scheme could be extended to individuals aged 45–59 for health checkups by easing the financial barrier, and show the importance of involving government recommendations and employer-purchased insurance.

## 1. Introduction

Screening has been widely recognised as a simple and effective measure for early diagnosis of most common chronic conditions, such as hypertension, hyperlipidemia, and diabetes [1,2,3]. Early detection is usually followed with timely diagnoses and interventions which prevents the onset or development of respective diseases, enhancing patients’ general health and quality of life. An article published by the Centers for Disease Control and Prevention (CDC), for instance, proved that early detection and treatment of type 2 diabetes was cost-effective in minimizing the incidences of major cardiovascular events, thereby increasing life-years and Quality Adjusted Life Years (QALYs) [4]. Costs imposed on the health systems by chronic illnesses treatments could, on the other hand, be reduced, which promotes fiscal sustainability on a macro level. According to the CDC, an expenditure of more than 3.2 trillion USD, for instance, was incurred by chronic and mental diseases in 2019 in the United States [5]. A recent study suggested that individuals aged 45 or above were six times more likely to develop any chronic conditions as compared to younger individuals [6]. The health concerns of the middle-aged are often out of the public’s attention, where a higher policy agenda usually goes to the health of youths and older adults with a vast variety of preventive programmes. The health needs of the middle-aged should be prioritised before the epidemic of chronic diseases overwhelms our health systems in a decade or two.

Nevertheless, there are a range of studies that have evaluated the predictors and barriers of health checkups across the globe. External barriers encompassing financial costs; time constraints; limited access to physicians; as well as internal factors such as perceived benefits and harms, and aversion to preventive medicine were identified in a myriad of studies. Healthcare vouchers, as a form of the financial lever and demand-side incentive, are adopted to encourage behavioural changes to popularise access to preventive care that is usually dominant in the private sector. For instance, India and Bangladesh offered maternal and reproductive service vouchers, whereas Japan implemented a free-coupon cervical screening programme [7,8]. We see that there is great potential for healthcare vouchers to fill the gap of service access for underserved populations, and it is a novel approach when compared to direct service provision by the public sector. Hence, there is an increasing demand for evidence to further investigate the relationship between care utilisation and voucher usage, yet the findings of this study should be generalised in other jurisdictions with caution due to cultural and contextual differences.

The Elderly Health Care Voucher Scheme (EHCVS) is a demand-side tool under the Public-Private Partnership Scheme (PPP) in Hong Kong, which was first introduced in 2009 as a fiscal incentive to promote primary care among the elderly [9]. The primary aims of the scheme are to promote the use of private healthcare services, especially in the prevention and management of chronic diseases, and to also reinforce the concept of family medicine. Vouchers with an annual amount of HKD2000 are automatically deposited to the voucher accounts of eligible citizens, who are Hong Kong permanent residents aged 65 or above, and they only have to present their Hong Kong Identification Cards to enrolled medical practitioners when they decide to purchase the service with vouchers. They can also decide to spend all the vouchers in one or multiple purchases, whilst there is a HKD8000 accumulation limit for each account [9]. A considerable variety of health services is offered where the elderly can purchase from up to nine medical professions. Hong Kong employs a dual-track healthcare system, where 87% of the inpatient services are provided by the public sector, and the private sector takes up 68% of outpatient services [6]. It leads to an extensive waiting time and exceedingly high bed occupancy rates at public hospitals, which could raise issues concerning the sustainability of the healthcare system in the future [10,11]. To expand the capacity of the healthcare system, EHCVS is designed to diverge cases of elderly patients to the private sector, which ensures equitable access to health services for most members of society. Several improvements have been made to the scheme over the years, encompassing an enormous increase in amount, from HKD250 to HKD2000 annually, and lowering the eligible age from 70 to 65 years old [9]. Despite the enhancements made, a variety of studies have revealed that the intended goals of the EHCVS have not been attained [12,13]. Although the utilization rate of the vouchers has reached 94.7%, the changes in health-seeking behaviours of the elderly deviated from our expectations. More respondents were utilizing both public and private healthcare services after the introduction of vouchers, and there were fewer individuals attending either public or private health services [12]. The majority of respondents from another study indicated that the major usage of vouchers fell on acute or episodic services, whilst only a very limited proportion of people purchased preventive or management services with their vouchers [13].

In response to the growing concern about the prevalence of chronic diseases among the middle-aged population and their profound demographic differences compared to the elderly, it is necessary to examine the public acceptance of well-defined vouchers for general health examinations. Owing to recent debates on expanding the current healthcare voucher scheme to those aged between 60–64 years, the study emphasises the impacts of the well-defined vouchers on those aged 45–59 instead [14]. Thus, this study is testing the hypothesis of whether an expansion of the current health care voucher policy could potentially exert an impact on general health checkup attendance among the middle-aged population. We examined the factors associated with uptake of checkups based on the health belief model. The amendment was based on an evaluation of EHCVS, suggesting that vouchers are more likely to promote health behaviour that is time-limited, simple, and well defined [15]. The findings could therefore contribute to the discussion on service provisions through a demand-side financing tool and offer insights to the government for further policy formulation.

## 2. Materials and Methods

### 2.1. Study Design

A cross-sectional online survey was conducted to investigate how likely annual preventive vouchers of HKD2000 would encourage Hong Kong citizens aged 45–59 to attend regular health checkups. All Hong Kong permanent residents aged between 45 to 59 and able to comprehend traditional Chinese were eligible. Ethics approval was obtained from The Survey and Behavioural Research Ethics Committee of The Chinese University of Hong Kong.

### 2.2. Data Collection

Data were collected through convenience sampling based on an online survey platform, consisting of an information cum consent form and questionnaire. They were disseminated via hyperlinks through various instant messaging applications, for instance, WhatsApp and Signal. These platforms are commonly used in Hong Kong and hence could maximise the opportunity of reaching the targeted population. The individuals would be directed to the questionnaire once they agreed to participate in the study by checking the box under the consent form.

### 2.3. Sample Size


$$n=\frac{Z_{\alpha /2}{}^{2}P\left(1-P\right)}{d^{2}}\;$$

Sample size was calculated with a formula where the proportion of health checkup uptake due to the vouchers was assumed as 50%, which resulted in the largest sample size because no similar local studies have been conducted. We set the precision level as 5% ($Z_{\alpha /2}Z_{\alpha /2}$ = 1.96, *P* = 0.5, *d* = 0.05), resulting in a minimum sample size of 385 (*n* = 384.16).

### 2.4. Questionnaire Design

A questionnaire was developed based on a conceptual model [16]. A total of 25 items were included in the survey under 4 sections, namely the attitudes and perceptions on health checkups and vouchers, Health Belief Model, healthcare service utilisation patterns, and socio-demographics. 

The first section focused on the hypothetical practices of the subjects if preventive vouchers were given to the respondents. They were asked about the likelihood of taking up the checkup habits after the introduction of the vouchers, rating on a 4-point Likert scale (1: Very Unlikely; 2: Unlikely; 3: Likely; 4: Very Likely). The second section was structured by the five constructs of the Health Belief Model, namely perceived susceptibility, perceived severity, perceived barriers, perceived benefits, and cues to action [16]. The model was adopted as it is a common and effective tool in predicting individuals’ health behaviours, and it has been widely used in various predictions of behaviours in preventive care [17,18,19]. Items were rated on a 4-point Likert scale as well. The healthcare service utilisation pattern and sociodemographic information were also collected under the fourth and fifth sections as potential predictor variables.

### 2.5. Data Analysis

Data collected were cleaned, encoded, and entered into the IBM SPSS Statistics for Windows, Version 25.0 (IBM Corp.: Armonk, NY, USA). The confidence level was set at 95%, and *p*-values less than 0.05 indicated significant associations. Descriptive analyses were first conducted to provide statistical backgrounds of the sample, whilst demographical data were compared with the general Hong Kong population to assess the representativeness of the sample. The primary objective was evaluated by a sum of percentages of respondents indicating “Likely” and “Very Likely” in the item, inquiring about the chances of attending health checkups with preventive vouchers. To facilitate the understanding of the data and outcomes, responses of “Likely” and “Very Likely” are grouped into one option, and “Very Unlikely” and “Unlikely” are grouped into another. The second objective of identifying independent factors associated with uptake was assessed by a multiple logistic regression model. We first conducted Chi-squared tests to examine the associations between the outcome and independent variables, which are items under Health Belief Model, health conditions and behaviours, and demographics. Variables with *p*-values less than 0.2 in the univariate analyses were included in the multivariate regression model, as well as characteristics showing significant differences with the general population, to minimise the possible bias of convenience sampling. Through backward stepwise selection, the variables with *p*-values less than 0.05 were concluded as significant variables for the decision of attending health checkups, with demographic factors controlled.

## 3. Results

### 3.1. Participant Characteristics

A total of 278 responses were collected from June to September 2021, with a completion rate of 99.6%. The age groups of the respondents were evenly distributed, and the proportions of those receiving financial aid, reporting hypertension and diabetes mellitus, and being employed were similar to those of the general population (Table 1). However, there were significantly fewer males, respondents with an educational level at primary or below, and individuals with a monthly income of less than HKD20,000. There were more respondents reporting hyperlipidemia when compared with the general population (*p* < 0.05). The mentioned variables were included in the multivariate regression analysis model for adjustment.

### 3.2. Likelihood of Respondents Attending Health Checkups with Vouchers

**Figure 1:**
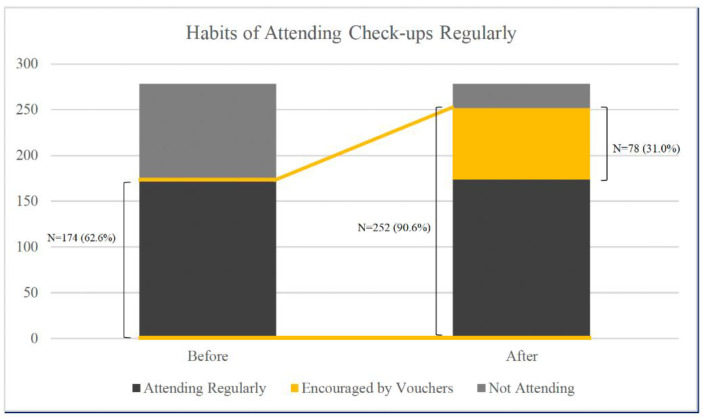
Percentage of respondents who attend regular checkups currently and plan to attend after the introduction of vouchers.

The proportion of respondents expressing “Likely” or “Very Likely” to attend health checkups with preventive vouchers of HKD2000 reached 90.6% (Figure 1). The proportion of votes for “Very Likely” and “Likely” was 61.1% and 29.5%, respectively. Among 278 respondents, 62.6% (174) had a current habit of health checkups, and a total of 90.6% (252) indicated that they were likely to purchase health checkups with well-defined vouchers, which showed an increase of 44.8% after the introduction of vouchers (78 of 174) when compared to the proportion with current attendance to regular health checkups.

### 3.3. Factors Associated with the Decision to Attend Health Checkups

The results of univariate analyses indicated that there were no significant associations established between the likelihood of attending health checkups with vouchers and most demographic factors except for education level (Table 2). More highly educated respondents were more likely to be encouraged, where those with secondary and post-secondary education backgrounds were six times (*p* = 0.028) and 10 times (*p* = 0.004)) more likely to attend checkups with vouchers, respectively. Respondents with hypertension and high cholesterol level were more likely to attend checkups with vouchers than other chronic diseases; however, no statistical significance was found. Among 17 Health Belief Model-related items, three items under cues to action showed strong positive significant associations (*p* < 0.05) with the likelihood of attending health checkups with voucher, including the government’s recommendation (*p* = 0.018), family and friends’ recommendation (*p* = 0.040), and full support by the employer’s insurance (*p* = 0.028). 

A multivariable logistic regression model (Table 3) was then constructed, with a total of 14 factors with *p*-values less than 0.2 in univariate analysis, including 10 variables from the Health Belief Model, and 4 factors for adjustment from socio-demographics and health conditions. Based on the health belief model, the perceived barrier of health checkups being costly (AOR 0.367, 95% C.I. 0.162–0.832, *p* = 0.016) was negatively associated with the likelihood, whilst the government’s recommendation (AOR 1.685, 95% CI 1.052–2.698, *p* = 0.030) and full support by the employer-purchased insurance (AOR 2.392, 95% CI 1.016–5.523, *p* = 0.041) were positively associated with uptakes of checkups.

## 4. Discussion

The findings suggested that more than 90.6% of the respondents were willing to attend health checkups with preventive vouchers given. The number of individuals willing to attend health checkups after the introduction of vouchers has increased by 44.8%, implying that the scheme has encouraged close to half of the individuals that currently do not have the habit to purchase health checkup services. The results imply that financial incentives could be an effective measure in promoting a particular preventive health behaviour, which echoes the findings from a range of studies shedding light on the effectiveness of demand-side financial incentives on the promotion of preventive services, including screening and vaccination [20,21].

The study has identified several significant factors under the Health Belief Model associated with the decision of attending health checkups with vouchers. Our findings show that the greater the extent that the individuals agreed checkups were costly, the less likely they were in taking up the habit, providing grounds for introducing a financial incentive to promote the behaviour. The average annual out-of-pocket (OOP) expenditure on health per person in Hong Kong is around HKD7500, while the market price for a regular health checkup is around HKD3000, or 40% of the current OOP expenditure [22,23]. Moreover, if an individual had to fully finance his or her own checkup every year, the percentage of OOP would reach 37.5%, which is very close to the 40% threshold of Catastrophic Health Expenditure set by the World Health Organisation [24]. Health checkups are generally not regarded as necessary by the general public when compared with acute episodic care for physician-diagnosed diseases; therefore, they usually lack the motivation to attend health checkups regularly. Results of health checkups do not always guarantee an enhancement in health, especially when there are no abnormalities detected in the test, and hence, some may see taking up health checkups as a waste of money [25]. The results of the study are, on the other hand, is in agreement with findings from a study examining OOP costs as a barrier to accessing colorectal screening in the United States [26].

Nevertheless, if the costs of health checkups were fully supported by employer-purchased insurance, the respondents are more likely to take up the habit. The finding is in agreement with the results from another local study regarding colorectal screening, which highlighted the importance of insurance coverage in promoting screening in the city [27]. Citizens would take up regular checkups as free services if no extra costs were incurred for the tests. There was a notable difference in significance in the association of the outcome with private insurance compared to insurance purchased by employers, where the degree of motivation of respondents may differ due to the self-financed nature.

The recommendations and promotions from the government were found to be another potential contributing factor to the uptake of health checkups. There is a lack of similar studies addressing and evaluating the role of government in promoting health checkups in the community. This is a compelling finding as the government stood out from the other commonly known possible key players in health promotion (for example, doctors, family and friends). This could be attributed to the credible and neutral nature of the government; people usually expect that the government would only give necessary advice. Moral hazards might have undermined the credibility of doctors, as private practitioners dominate the primary care sector in Hong Kong; they may as well be the service providers while recommending that the patients to undergo checkups. Patients may be concerned about moral hazards if the procedure is necessary, whilst there is no conflict of interest if it is promoted or recommended by the government [28]. On the other hand, the government is usually in a neutral position to offer more reliable and trustworthy information when compared to friends and family members. The role of the government in changing individuals’ health behaviours is yet to be determined. Nevertheless, the present findings suggest that a top-down approach from the government of introducing the defined healthcare vouchers could enhance uptake of health checkups among the middle-aged.

Although our findings do not indicate any significant differences among the groups in age, sex, and monthly individual income, a few studies have shown that these characteristics may influence the intentions for checkups [29,30]. Thus, further studies would be suggested to explore the relationships of these demographic factors and health checkups. If more than one factor affects the decision, adapting a multi-dimensional approach may be beneficial to reduce hesitancies and maximise the screening uptake [31].

### 4.1. Strengths and Limitations

This is one of the very few studies exploring how vouchers, as a demand-side financial incentive tool, could contribute to the promotion of preventive care in the middle-aged population. Nevertheless, there are several limitations of the research. First and foremost, due to the cross-sectional nature of the study, the results could not be used as evidence for causality. Second, as convenience sampling was adopted as the major sampling method, the sample skews towards female, well-educated, and high-income groups, yet the effects of the variables were adjusted by including them in the multiple logistic regression model. Lastly, this is a small-scale study with a sample size of 278 subjects; the generalisability of the findings is therefore limited. However, after reapplying *p* = 0.906 into the proportion formula, the required sample size for the study is 131, which is less than the current sample size.

### 4.2. Recommendations

Future studies could further examine the relationship of attending health checkups with other health behaviours, for instance, smoking, alcohol drinking, and level of physical activity, as the level of health consciousness could be a determining predictor [32]. Demographic factors, encompassing socio-economic status and education level, could be further explored as well to outline the effect of health literacy and self-reported health status on the decision of attending health checkups regularly [33,34]. It is also noteworthy that education disparities also contribute to the habit of attending checkups, as shown in the univariate analyses. Enhancing the usage of vouchers in particular groups, for instance, those who are less educated, could be a target in future research, health education, and policy intervention to minimize health disparities.

## 5. Conclusions

We identified financial concerns as a major barrier to promoting health behaviours. The study suggests that extending the voucher scheme from the population aged 65 or above to those aged 45–59 and limiting it to a well-defined service type of preventive care is effective in encouraging the middle-aged to attend regular health checkups, which can detect chronic and life-threatening health conditions early, promoting the chance of cure and limiting the risks of complications. The Government should work on the formulation of relevant policies to improve the overall health of the middle-aged population, which is a high-risk group in developing chronic and life-threatening conditions, in the form of demand-side financial tools, as well as alleviating the burden on the healthcare system in the long run.

## Figures and Tables

**Table 1:** Participants Characteristics (N = 278).

	Hong Kong Population ^a^ (%)	Study Sample N = 278, *n* (%)	*p*
**1.** **Age**			0.972
45–49	(30.9)	84 (30.2)	
50–54	(35.1)	101 (36.3)	
55–59	(34.0)	93 (33.5)	
**2.** **Gender**			0.007 **
Male	(45.8)	84 (30.2)	
Female	(54.2)	194 (69.8)	
**3.** **Education Level**			<0.001 **
Primary or below	(24.2)	7 (2.5)	
Secondary	(53.0)	118 (42.4)	
Post-Secondary	(22.8)	153 (55.0)	
**4.** **Monthly Individual Income**			<0.001 **
No Income	(49.0)	83 (29.9)	
Less than HKD20,000	(33.0)	67 (24.1)	
HKD20,000 or more	(17.0)	128 (46.0)	
**5.** **Self-reported Longstanding Health Conditions** ^b^			
Yes	(32.1)	97 (34.9)	0.45
Hypertension	(12.2)	52 (18.7)	0.171
Hyperlipidemia	(6.1)	53 (19.1)	0.005 **
Diabetes	(4.2)	26 (9.4)	0.268
**6.** **Receiving Government Aids**			1.00
Yes	(5.1)	14 (5.0)	
No	(94.9)	264 (95.0)	

Notes: **: *p* < 0.05; ^a^ 2016 Population By-Census—Summary Results. Census and Statistics Department HKSAR, 2017.; ^b^ Thematic Household Survey Report No. 68. Census and Statistics Department HKSAR, 2019.

**Table 2:** Possible confounding factors associated with likelihood of attending health checkups with vouchers by univariate analyses.

Factors	*n*	Likelihood %	COR	95% CI		*p*
**Socio-demographics**						
**Age**						
45–49	84	90.5	Reference			
50–54	101	94.1	1.667	0.554	5.010	0.363
55–59	93	87.1	0.711	0.275	1.833	0.480
**Gender**						
Male	84	88.1	Reference			
Female	194	91.8	1.503	0.652	3.466	0.339
**Monthly Individual Income**						
No Income	109	91.7	Reference			
Less than HKD20,000	41	87.8	0.922	0.304	2.801	0.886
HKD20,000 or more	128	90.6	0.985	0.615	1.576	0.949
**Receiving Government Aids**						
No	264	90.1	Reference			
Yes	14	85.7	0.600	0.127	2.840	0.520
**Education Level**						
Primary	7	57.1	Reference			
Secondary	118	90	6.058	1.218	30.125	0.028 **
Post-Secondary	153	93.5	10.725	2.105	54.654	0.004 **
**Health Conditions and Behaviours**						
**Self-reported Longstanding Health Conditions ^1^**						
Yes	97	92.8	1.508	0.611	3.724	0.373
Hypertension	52	94.2	1.851	0.534	6.414	0.332
Hyperlipidemia	53	94.3	1.898	0.548	6.573	0.312
Diabetes	26	88.5	0.770	0.215	2.762	0.688
**Habit of Attending Health Checkups**						
No	104	91.3	Reference			
Yes	174	90.2	0.875	0.375	2.041	0.757
**Health Belief Model**						
**Perceived Susceptibility**						
Overall susceptibility			0.799	0.491	1.300	0.365
**Perceived Severity**						
Severe consequences			1.205	0.752	1.932	0.438
Health and financial burden			1.217	0.710	2.087	0.475
**Perceived Benefits**						
Detect chronic diseases early			1.578	0.844	2.953	0.153 *
Lower severity of diseases			1.425	0.745	2.726	0.285
**Perceived Barriers**						
Costly			0.517	0.254	1.051	0.068 *
Takes time			0.696	0.431	1.124	0.138 *
Does not match their schedule			0.716	0.446	1.150	0.167 *
Geographically inconvenient			0.647	0.411	1.018	0.060 *
Side effects			0.841	0.539	1.314	0.447
**Cues to Action**						
Government’s recommendation			1.708	1.098	2.658	0.018 **
Family and friends’ recommendation			1.725	1.024	2.905	0.040 **
Doctor’s recommendation			0.807	0.39	1.672	0.564
Having chronic conditions			1.060	0.468	2.402	0.889
Close relatives suffering from chronic conditions			1.648	0.979	2.774	0.060 *
Fully covered by private insurance			1.659	0.858	3.207	0.133 *
Fully covered by insurance purchased by employers			2.100	1.082	4.075	0.028 **

Notes: COR—Crude Odds Ratio; CI—Confidence Interval; HCVS—Health Care Voucher Scheme; * *p* < 0.2; ** *p* < 0.05; ^1^ The 3 most diagnosed chronic diseases in Hong Kong have been separately analysed.

**Table 3:** Factors associated with likelihood of attending health checkups with vouchers by multivariate logistic regression.

Factors	AOR	95% CI		*p*
**HBM**				
**Perceived Barriers**				
Regular checkups are costly	0.367	0.162	0.832	0.016 **
**Cues to Action**				
Government’s recommendation	1.685	1.052	2.698	0.030 **
Fully covered by insurance purchased by employers	2.395	1.036	5.523	0.041 **

Notes: AOR—Adjusted Odds Ratio; CI—Confidence Interval; HBM—Health Belief Model; ** *p* < 0.05.

## Data Availability

The data presented in this study are available on request from the corresponding author. The data are not publicly available due to them containing information that could compromise the privacy of research participants.
